# A Hybrid Feature Selection Approach to Screen a Novel Set of Blood Biomarkers for Early COVID-19 Mortality Prediction

**DOI:** 10.3390/diagnostics12071604

**Published:** 2022-06-30

**Authors:** Asif Hassan Syed, Tabrej Khan, Nashwan Alromema

**Affiliations:** 1 Department of Computer Science, Faculty of Computing and Information Technology Rabigh (FCITR), King Abdulaziz University, Jeddah 22254, Saudi Arabia; nalromema@kau.edu.sa; 2 Department of Information Systems, Faculty of Computing and Information Technology Rabigh (FCITR), King Abdulaziz University, Jeddah 22254, Saudi Arabia; tkamin@kau.edu.sa

**Keywords:** COVID-19, blood biomarkers, hybrid-feature selection, filter-based feature selection, two-tailed unpaired *t*-test, meta-heuristic method, machine learning models, mortality risk prediction

## Abstract

The increase in coronavirus disease 2019 (COVID-19) infection caused by severe acute respiratory syndrome coronavirus 2 (SARS-CoV-2) has placed pressure on healthcare services worldwide. Therefore, it is crucial to identify critical factors for the assessment of the severity of COVID-19 infection and the optimization of an individual treatment strategy. In this regard, the present study leverages a dataset of blood samples from 485 COVID-19 individuals in the region of Wuhan, China to identify essential blood biomarkers that predict the mortality of COVID-19 individuals. For this purpose, a hybrid of filter, statistical, and heuristic-based feature selection approach was used to select the best subset of informative features. As a result, minimum redundancy maximum relevance (mRMR), a two-tailed unpaired *t*-test, and whale optimization algorithm (WOA) were eventually selected as the three most informative blood biomarkers: International normalized ratio (INR), platelet large cell ratio (P-LCR), and D-dimer. In addition, various machine learning (ML) algorithms (random forest (RF), support vector machine (SVM), extreme gradient boosting (EGB), naïve Bayes (NB), logistic regression (LR), and k-nearest neighbor (KNN)) were trained. The performance of the trained models was compared to determine the model that assist in predicting the mortality of COVID-19 individuals with higher accuracy, F1 score, and area under the curve (AUC) values. In this paper, the best performing RF-based model built using the three most informative blood parameters predicts the mortality of COVID-19 individuals with an accuracy of 0.96 ± 0.062, F1 score of 0.96 ± 0.099, and AUC value of 0.98 ± 0.024, respectively on the independent test data. Furthermore, the performance of our proposed RF-based model in terms of accuracy, F1 score, and AUC was significantly better than the known blood biomarkers-based ML models built using the Pre_Surv_COVID_19 data. Therefore, the present study provides a novel hybrid approach to screen the most informative blood biomarkers to develop an RF-based model, which accurately and reliably predicts in-hospital mortality of confirmed COVID-19 individuals, during surge periods. An application based on our proposed model was implemented and deployed at Heroku.

## 1. Introduction

### 1.1. Rationale for Developing COVID-19 Mortality Risk Prediction Technique

COVID-19 presents a broad spectrum of clinical manifestations, ranging from asymptomatic to critically ill COVID-19 individuals, with progressive respiratory failure [1,2,3,4,5,6,7]. At this pandemic stage, an unexpected increase in COVID-19 cases has placed immense pressure on health care services, leading to a shortage of intensive care resources. Most of the individuals admitted to hospitals will survive, but some individuals develop severe respiratory failures requiring ventilators. In addition, many of these individuals on ventilators succumb to their rapidly progressive respiratory dysfunctions. Identifying crucial predictive blood biomarkers of mortality from COVID-19 individuals would be invaluable for understanding the relative risk of death when triaging individuals for hospital admission. Moreover, identifying key biomarkers of mortality provides guidance in the form of a policy decision for allocating scarce resources to individuals requiring immediate respiratory monitoring and at imminent risk of death. In this context, ML can assist by examining big data with numerous features to rapidly discover patterns and build ML models that accurately assess risk factors associated with the severity of COVID-19 infection. ML techniques have played a significant role in identifying COVID-19 individuals using chest X-rays and computer tomography images [8,9,10].

Moreover, extensive research has been carried out on deep learning (DL) for image classification during the last 2 to 3 years of the COVID-19 pandemic [11]. However, a deep neural network’s architecture influences the performance of DL methodology [12]. Therefore, over the past 2 years of the pandemic, the use of meta-heuristics in optimizing the architecture and parameters of the deep neural network has gained significant popularity [13]. Furthermore, due to their simplicity, flexibility, and problem independence, researchers used meta-heuristics to solve various non-linear optimization problems [14,15]. Yet, image-based modality is challenging in the diagnosis of COVID-19 individuals in a low resource environment [16,17]. As a result, scalable solutions must be developed based on alternative data acquired using inexpensive and easily accessible tests. Various ML models have been built in this context to predict the risk for progression to severe COVID-19-related complications and mortality [18,19,20,21,22,23,24,25,26,27,28,29,30,31,32]. The development of ML models is significantly relevant in a situation with limited resources compared with the sharp increase in the number of COVID-19 individuals. Resource allocation depends on distinguishing admitted individuals based on their prognosis. In addition, it is an important issue as the severity of cases places pressure on medical services, causing a shortage of intensive care resources in low to medium settings. Unfortunately, there are no clinically approved prognostic blood markers to differentiate individuals that require immediate medical care and assess their associated mortality. Therefore, the capacity to distinguish COVID-19 cases that are at imminent risk of mortality has become an urgent yet challenging necessity.

### 1.2. Review of Literature

In this regard, different routine blood test-based ML models have shown promise in severity and mortality prediction. Table 1 summarizes the work on implementing the blood biomarker-based machine learning techniques for predicting mortality risk.

For example, Banerjee et al. [33] used complete blood counts to identify COVID-19 individuals rather than traditional screening based on clinical manifestations. The authors discovered that positive COVID-19 cases display lower amounts of lymphocytes, platelets, and leukocytes. Brinati et al. [34] used routine blood biomarkers to predict COVID-19 cases from a sample of 279 COVID individuals by training and testing various ML classification algorithms, which resulted in the identification of positive COVID-19 cases with recall ranging between 92 and 95% and an accuracy ranging between 82 and 86%. Thell et al. [35] used standard blood laboratory biomarkers and their respective values as a clinical decision tool to distinguish negative and positive SARS-CoV-2 individuals. In addition, Yang et al. [36] assessed the implementation of ML algorithms in standard laboratory blood tests to predict positive COVID-19 cases that offered a chance to detect COVID-19 infection in areas where reverse transcriptase assay for screening of positive COVID-19 cases is unavailable. Moreover, an ML-based model was employed to predict the mortality and severity stages of COVID-19 individuals. Rahman et al. [37] employed readily accessible complete blood count (CBC) attributes to predict the severity of infection of COVID-19 individuals, and the classification model was validated using an additional external dataset exhibiting high predictive accuracy. In addition, Chowdhury et al. [38] explored clinical representations and demographic features and the response outcome to identify critical clinical and demographic biomarkers, such as lymphocytes, neutrophils, hs-CRP, LDH, and variable information acquired during patient admission to the hospital, as well as age to predict the individual patient’s mortality using multi-tree XGBoost model. Furthermore, Chowdhury et al. [38] developed a nomogram that predicts the mortality risk among confirmed COVID-19 cases. First, an integrated score was computed with the corresponding patient’s death probability. Then, COVID-19 individuals were divided into low, moderate, and high-risk groups. The AUC of the training and validation study nomogram was 0.961 and 0.991, respectively. Vaid et al. [39] proposed, based on the XGBoost feature importance and XGBoost classifier, that increased LDH level, hyperglycemia, acute kidney damage, higher age, CRP, and anion gap play a critical role in predicting critical events in individuals with COVID-19 infection, as well as mortality. Aladag and Atabey [40] have tried to predict the mortality risk for critically ill COVID-19 individuals by employing coagulopathy biomarkers. Terwangne et al. [41] presented the predictive accuracy of a model based on Bayesian network analysis for severity classification of COVID-19 individuals using five crucial clinical features, namely age, acute kidney injury, lymphocytes, APTT, and LDH. Huang et al. [42], in a retrospective analysis of 336 positive COVID-19 individuals and 139 negative control, applied nine independent clinical risk factors during patient admission to compute their risk scores and distinguish them into different risk categories. With the use of LR, Cia et al. [43] assessed the independent relationship between the baseline level of four clinical parameters, such as LDH, NLR, D-dimer, and CT score on admission and the severity of COVID-19 infection. A high level of NLR and LDH could assist in detecting positive COVID-19 cases in the high-risk groups. Moreover, the model’s sensitivity showed enhancement when LDH and NLR were used together. Liu et al. [30], using a combination of CRP and NLR, could predict 3-day disease severity in only 84 hospitalized COVID-19 individuals diagnosed with pneumonia. Zhang et al. [31] used univariate and multivariate logistic regression analysis to select white blood cell count, age, neutrophil, myoglobin, and glomerular filtration rate for building a scoring system that predicts severity of COVID-19 individuals. The model was validated on external validation data with 22 COVID-19 individuals. Shang et al. [32] built a scoring system to distinguish COVID-19 individuals into low- and high-risk groups. The high-risk group of individuals had a significantly higher chance of mortality than those in the low-risk group. Eight independent variables, including blood variables and age, were selected using multivariable analysis and lasso binary logistic regression coefficients. A scoring model was built using the blood variable and age, then it was validated in an independent validation cohort that could successfully discriminate between the two classes with an AUC of 0.938 (95% Cl, 0.902–0.973). Wang et al. [44] developed two predictive models using laboratory and clinical attributes to predict the mortality of COVID-19 individuals admitted to the hospital. The laboratory model built using features, such as neutrophil and lymphocyte count, peripheral capillary SpO2, AST, D-dimer, hs-CRP, GFR, and age proved to be of more discriminative power with an AUC of 0.88 (95% CI, 0.75–0.96) on an independent cohort validation dataset (*n* = 44). Xie et al. [45] screened LDH, age, SpO2, and lymphocyte count, as a subset of essential attributes to generate a model for mortality prediction. Later, the authors validated their model on an independent validation cohort with an AUC of 0.98. Furthermore, they established a nomogram to compute the probability of mortality using their mortality prediction model. Jimenez-Solem et al. [26] used conventional risk factors, namely BMI, age, and hypertension, to build a mortality prediction model from the COVID-19 data from the United Kingdom and Denmark, which showed a higher discriminative power with an AUC of 0.818 on hospital admission, 0.906 at diagnosis, and 0.721 during ICU admission. Bolourani et al. [24] developed the XGBoost model using the most influential variables, such as emergency severity index (ESI) level, age, respiratory rate, serum lactate, demographic characteristics, and the type of oxygen delivery used in the emergency department to predict respiratory breakdown within 48 h of admission for COVID-19 confirmed patients. The XGBoost model performance was better, with an AUC of 0.77 and a mean accuracy of 0.919. Karthikeyan et al. [46] developed a machine learning-based clinical decision support system using blood test data for an earlier COVID-19 mortality prediction. The XGBoost feature importance method was used to screen the five most informative features: Lymphocytes, neutrophils, hs-CRP, LDH, and age. The features obtained using XGBoost-based feature importance were used to build a neural network classification algorithm-based model that assists in predicting mortality with 96% accuracy as early as 16 days before the outcome. Yan et al. [47] proposed an interpretable single tree XGBoost model using the three most informative features, LDH, hs-CRP, and lymphocytes, for COVID-19 mortality prediction with a 94% accuracy as early as 3 days before the patient outcome.

Although there have been recent works utilizing ML approaches to screen informative blood biomarkers, and classification and scoring models to predict the COVID-19 confirmed patient outcome and severity analysis. Yet, the proposed model’s accuracy and precision in recent literature are not enough for use as a clinical biomarker in the accurate and reliable prediction of COVID-19 patient outcomes, as well as for looking into the untapped potential of meta-heuristics to handle high-dimensional data in COVID-19 datasets. Therefore, there is still scope in exploring the most crucial subset of blood biomarker from a high dimensional blood biomarker-based COVID-19 clinical dataset that can predict the COVID-19 outcome (mortality) with greater accuracy and precision, thereby understanding the relative risk of death of COVID-19 individuals on admission to hospital. As a result, in the present study, various feature selection approaches (filter, statistical, and meta-heuristics), classification algorithms, and robust testing were employed to screen the most reliable and accurate blood biomarker that can precisely predict the outcome of COVID-19 individuals during the early stages.

Therefore, by screening reliable blood biomarkers that assist in predicting COVID-19 post-infection outcome, we can save many people at imminent risk of death during this pandemic. As a result, the novelty of the current study can be stated as the screening of reliable blood biomarkers using a hybrid feature selection technique to build an ML-based model that accurately predicts the outcome of positive COVID-19 individuals with fewer features and higher confidence and reliability. In addition, the healthcare services can use our model to precisely enhance treatment policy by appropriately utilizing the limited health care and life support resources in developing and underdeveloped countries [33,34,36,45]. Furthermore, the present study adds to the knowledge of developing mortality risk prediction techniques using ML approaches.

The rest of this paper is organized as follows: Section 2 discusses the study’s methodology by describing the datasets used in this paper, the details of data preprocessing, hybrid feature selection approaches and stages for machine learning classifiers, and model generation for predicting the outcome of COVID-19 individuals. Section 3 discusses the result of the feature selection and the classification models. Section 4 discusses the result and validates the performance against available ML models for COVID-19 patient outcome prediction. Finally, the article is concluded in Section 5.

## 2. Materials and Methods

### 2.1. Machine Learning Pipeline

**Figure 1:**
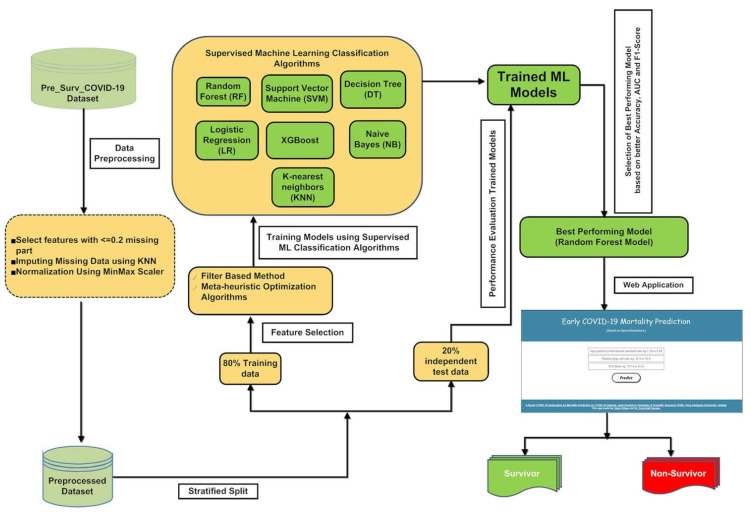
A pictorial representation of the ML pipeline for implementing an application that predicts the mortality of positive COVID-19 cases.

The overall pipeline for building a machine learning-based application to perform mortality prediction in hospitalized individuals is depicted in Figure 1. Our study trained and tested the classification models using the final day (discharge or death day) samples of every confirmed COVID-19 patient admitted to hospital. Subsequent data preprocessing, a hybrid feature selection approach using a filter-based feature selection method, namely, mRMR, a two-tailed unpaired *t*-test, and four state-of-the-art meta-heuristic methods were used to identify the most relevant subset of features for building classification models that predict mortality of COVID-19 individuals admitted to hospital for treatment. The four optimal feature subset obtained using the meta-heuristic algorithms were trained using stratified five-fold cross-validation on various supervised classification algorithms to build supervised ML models. The trained models’ predictive ability and statistical significance were tested using stratified five-fold cross-validation on an independent dataset. The ML model’s performance was evaluated and assessed based on different statistical performance matrices whose average and standard deviation are reported in the article. The step-by-step model building, evaluation, and implementation process are documented in the following methodology sections.

### 2.2. Dataset and Preprocessing

The Pre_Surv_COVID_19 data were used for feature selection, and building classification models were obtained from [47]. The Pre_Surv_COVID_19 dataset is a time series of COVID-19 patient data collected at different time intervals from Tongji Hospital in Wuhan, China. The initial set of time series data comprising data from 375 COVID-19 validated or suspected individuals with 34 biomarkers (attributes) alongside data collection time, admission time, discharge time, and response variable (survivor or non-survivor) was used for training ML models. Most of the samples in the initial dataset had multiple readings taken at different points of time during the COVID-19 patient’s stay at the hospital. However, we have used the final reading of the attributes as data for each sample to train and test the models.

The data of the final sample were taken as an input model to screen features that access the crucial biomarkers for disease severity and distinguish individuals that require immediate medical attention. Biomarkers (attributes) in training test data with a missing part ≤ 0.20 (missing part = missing count/total samples) were dropped and not employed for further analysis. The KNN algorithm imputed the missing values in training and test datasets. The imputed values were calculated by averaging the values of 10 nearest neighbors, and weighing by the inverse of the squared Euclidean distance. Min–Max scaling algorithm was used to normalize the Pre_Surv_COVID_19 dataset. Since certain features in the COVID-19 dataset were not normally distributed, thus the Min-Max scaling algorithm was used to normalize the distribution of the datapoint in those features. After preprocessing the data, the COVID-19 dataset consisted of 54 attributes, including the class and 335 samples (174 individuals died and 201 individuals survived). The features in the preprocessed COVID-19 data were further processed based on two classes (survivors and non-survivors). Individuals who survived were encoded as ‘0’, and those who did not survive were encoded as ‘1’.

### 2.3. Partitioning of the Processed Data

The preprocessed dataset with 375 samples was randomly partitioned into an 80% training set, and the remaining 20% of the sample was selected for the independent testing set. The 80% training set consisted of 300 instances, of which 161 were survivors, and the remaining 139 were non-survivors. In addition, it is optimal to test the trained models on independent test data to avoid prediction bias. Therefore, 20% of testing data was used to evaluate the performance of the trained models built using various optimal combinations of features. The testing dataset consisted of 75 instances, where 40 were survivors, while 35 were non-survivors.

### 2.4. Evaluation Metrics

The predictive performance of the supervised ML models was evaluated using the following metrics:

#### 2.4.1. Accuracy and Confusion Matrix

Accuracy is an essential metric in assessing classification models only when the classes are balanced. In this study, the test dataset is not unbalanced. Therefore, accuracy provides a better insight into the model’s predictive performance. The model’s accuracy ranges from 0 to 1, where 1.0 is the best, while ‘0.0’ is the worst-performing model. Computationally, the accuracy is calculated using the following formula:

(1) $$Accuracy=\frac{TP+TN}{TP+TN+FP+FN}$$

In Equation (1), *TP*, *TN*, *FN*, and *FP* denote true positive, true negative, false negative, and false positive, respectively. The terminology from the confusion matrix is defined as follows:

- A positive class (in our study, the non-survivor);
- A negative class (in our research, the survivor);
- A true positive is a predicted outcome where the model correctly predicts the positive class for an actual positive instance (non-survivor) in our COVID-19 dataset;
- A true negative is an outcome where the model correctly predicts the negative class for an actual negative instance (non-survivor) in our COVID-19 dataset;
- A false positive is an outcome where the model incorrectly predicts the positive class;
- A false negative is an outcome where the model incorrectly predicts the negative class.

In addition, a confusion matrix can provide insight into the prediction models’ errors and error types (type I and II). The breakdown of the model’s correct and incorrect prediction compared with the actual outcome enables us to overcome the limitation of using accuracy alone as a statistical evaluator for assessing a classification model’s performance [48]. In addition, we aim to develop a mortality prediction model that focuses only on the positive class (non-survivors); other performance matrices, namely recall, precision, and F1 score, are more valuable than accuracy alone [49].

#### 2.4.2. F1 Score, Precision, and Recall

The F1 score is the harmonic mean of precision and recall, as follows:

(2) $$F1\;score=\frac{2\times \mathrm{Precision}\times \mathrm{Recall}}{\mathrm{Precision}+\;\mathrm{Recall}}$$

where recall and precision are calculated using the formulas shown in Equations (3) and (4), respectively, as follows:

(3) $$\mathrm{Recall}=\frac{TP}{TP+FN}$$

and

(4) $$\mathrm{Precision}=\frac{TP}{TP+FP}$$

#### 2.4.3. The Area under the Receiver Operating Characteristic Curve (ROC-AUC)

The value of AUC, which stands for the area under the receiver operating characteristic curve (ROC), provides a cumulative performance measure across various classification or decision thresholds, ranging from 0 to 1. The AUC value measures the complete two-dimensional area under the ROC curve beginning from (0,0) and finishing at (1,1), and thereby measures the precise model’s ability to distinguish the classes in the dataset. For example, a random classification model has an AUC value of 0.5, while a perfect classification model that can accurately distinguish between the classes in a dataset has an AUC value equal to 1. The ROC curve is a two-dimensional plot comprising two statistical evaluators; namely, true positive rate (*TPR*) and false positive rate (*FPR*), calculated using Equations (5) and (6), respectively, as shown below:

(5) $$TPR=\frac{TP}{TP+FN}\;$$

and

(6) $$FPR=\frac{FP}{FP+TN}$$

### 2.5. Proposed Hybrid Feature Selection Approach

**Figure 2:**
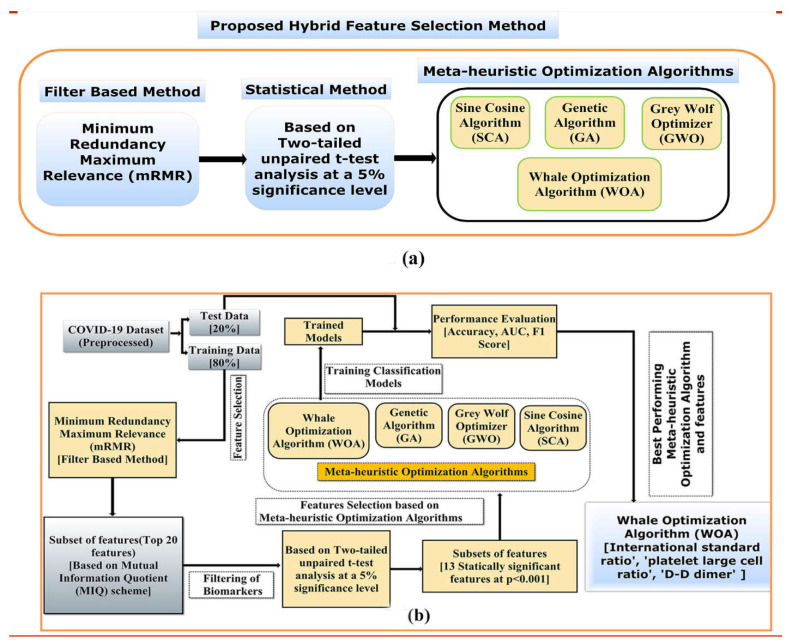
(**a**) Pictorial representation of the proposed hybrid feature selection method and (**b**) the workflow of the proposed hybrid feature selection method to screen the most informative ML model for predicting the mortality risk of COVID-19 individuals.

A hybrid filter-based feature selection method involving mRMR, a two-tailed unpaired *t*-test, and four state-of-the-art meta-heuristic methods were used to screen the most informative features for the COVID-19 mortality prediction task, as represented in Figure 2a. A pictorial illustration of the work flow of the proposed hybrid feature selection strategy, to identify the most relevant subset of features for training various supervised classification models that can predict the mortality of COVID-19 individuals with better accuracy and reliability, is represented in Figure 2b.

First, the mRMR method obtained feature importance based on the F-statistic with a correlation quotient (FCQ) scoring scheme. Eventually, a subset of the twenty most informative features was selected based on the FCQ score. Second, a two-tailed unpaired *t*-test at a 5% significance level was performed between the mean value of the distribution of the twenty most informative features screened through mRMR across two classes of population (COVID-19 survivor and non-survivor). Next, the two-tailed unpaired *t*-test was performed to screen features that show a significant difference between the mean values of the feature across the COVID-19 survivor and non-survivor population in the COIVD-19 dataset. Third, four meta-heuristic algorithms were used to select the optimal feature subset from the group of features whose mean distribution is significantly different between the two classes of population. Finally, the four optimal feature subset obtained using the meta-heuristic algorithms were trained using stratified five-fold cross-validation on supervised classification algorithms to develop supervised ML models.

#### 2.5.1. The mRMR Feature Importance

The mRMR, which stands for maximum relevance-minimum redundancy, is so-called due to the fact that at each iteration the algorithm selects features with a maximum correlation with the outcome variable and the most negligible correlation for the attributes selected in the prior iterations. Furthermore, the mRMR is a minimal-optimal feature selection algorithm, which indicates that the algorithm tends to select the smallest relevant subset of features for any specified ML assignment [50]. In the mRMR algorithm, at each iteration *i*, a score for each attribute (*f*) to be assessed is calculated using Equation (7), as shown below:

(7) $$score_{i}(f)=\frac{relevance(f|\mathrm{target})}{redundancy(f|features\;selected\;untill\;i-1)}$$

The best feature (*f*) selected at iteration *i* is the one with the highest score. Since our COVID-19 data are continuous, we use the F-statistics to measure the relevance of a feature at the *i*-th iteration with the outcome variable. The redundancy is estimated as the average Pearson correlation between the feature (*f*) at the *i*-th iteration and the features selected in the previous iterations. Therefore, Equation (7) can now be represented as follows:

(8) $$score_{i}(f)=\frac{F(f,\mathrm{target})}{\sum_{s\in \;features\;selected\;until\;i-1}\left|corr(f,s)\right|/(i-1)}$$

where *f* is the evaluated feature, *i* is the *i*-th iteration, *corr* is the Pearson correlation, and *F* is the F-statistic.

Moreover, the maximum relevance of a minimal-optimal feature set *S* for outcome variable *o* is computed by calculating the mean value of each F-test value between the individual feature *f* and the outcome variable *o*, and the equation is represented as follows:

(9) $$\max\;V_{F},V_{F}=\frac{1}{\left|S\right|}\sum_{i\varepsilon S}F(i,c)$$

The minimum redundancy of every feature in the minimal-optimal feature subset *S* is computed by estimating the mean value of each Pearson correlation between feature *a* and feature *b*, and the equation is represented as follows:

(10) $$\min\;W_{C},W_{C}=\frac{1}{{\left|S\right|}^{2}}\sum_{i,j\varepsilon S}\left|c(i,j)\right|$$

The Pearson correlation coefficient is depicted by c (a,b). In our study, the F-statistic with a correlation quotient (FCQ) was used as an mRMR optimization condition that relates the maximal relevance and minimal redundancy, as shown in Equation (11) below:

(11) $$\max(\frac{{}_{V_{{}_{F}}}}{{}_{W_{{}_{C}}}})$$

Our study selected the top 20 features (k = 20) as the feature size. The selected twenty features were further screened using a student *t*-test to screen features that significantly differ in frequency distribution in the target classes of population (survivor and non-survivor).

#### 2.5.2. Student *t*-Test

A two-tailed unpaired *t*-test [51] at a 5% significance level was performed between the mean value of the distribution of the 20 most informative features screened through mRMR across two classes of population (COVID-19 survivor and non-survivor). In addition, a subset of features demonstrating a significant difference between the mean values of the feature across the two classes of population were screened.

#### 2.5.3. Meta-Heuristic Selection

Four state-of-the-art meta-heuristic algorithms, namely, whale optimization algorithm (WOA), genetic algorithm (GA), grey wolf optimizer (GWO), sine cosine algorithm (SCA), were employed to screen the optimal global subset of features from the features set obtained using two-tailed unpaired *t*-test selection strategy. The four state-of-the-art meta-heuristic algorithms (WOA, GA, GWO, SCA) are discussed below.

**Whale Optimization Algorithm**

WOA is a meta-heuristic optimization algorithm inspired by the hunting mechanism of humpback whales in nature [52]. The algorithm involves two steps, search for prey and encircling the prey. The first stage of the WOA is explained as follows:

**Search for Optimum Search Solution**

The optimum solution is initially unknown. Therefore, the WOA algorithm assumes the current best solution as the target prey. After the best solution is defined, the other search agents update their position toward the best solution using the following equations:

(12) $$\vec{D}=\left|\vec{C}.{\vec{X}}_{p}(t)-\vec{X}(t)\right|$$

(13) $$\vec{X}(t+1)={\vec{X}}_{p}(t)-\vec{A}.\vec{D}(1)$$

Here, (*t*) represents the current iteration, *C* and *A* are the coefficients of the vector, *X* represents the current position vector of an agent (whale), and *Xp* is the position vector of the prey (initial optimum solution). Moreover, the vectors *A* and *C* are estimated as shown below:

(14) $$\vec{A}=2\vec{a}.r_{1}-\vec{a}$$

(15) $$\vec{C}=2.{\vec{r}}_{2}$$

Here, the parameter ‘*a*’ is linearly decreased from 2 to 0 over the maximum number of iterations to explore and exploit the search agent. Based on the value of the parameter ‘*p*’, the WOA can switch between a circular or spiral movement for the encircling prey.

**Encircling Prey**

Mathematically, the encircling of prey involves two optimization mechanisms depending on the value of parameter ‘*p*’.

1. Shrinking encircling mechanism

When the value of *p* < 0.5, then the new position of the agent (whale) is updated based on the equation shown below:

(16) $$\vec{X^{*}}(t)-\vec{A}\vec{D}\;p<0.5$$

The parameter ‘*p*’ value is any random number ranging from 0 to 1. The shrinking encircling mechanism is achieved when ‘*a*’, as shown in Equation (2), is decreased from 2 to 0 over maximum iterations.

2. Spiral updating position

The spiral updating position mechanism initially estimates the distance represented as ‘*D*’ between the agent (whale) located at (*X*, *Y*) and the prey located at (X*, Y*) and is calculated as follows:

(17) $$\vec{D^{\prime }}\;=\left|\vec{X^{*}}\left(t\right)-\vec{X}(t)\right|$$

Finally, when the value of *p* > 0.5, then the new position of the whale (best solution to date) is updated based on the spiral equation mimicking the helix-shaped movement of the humpback whales, as shown below:

(18) $$\vec{X}(t+1)=\vec{D^{\prime }}e^{bl}\cos(2\Pi l)+\vec{X^{*}}(t)$$

Here, ‘*l*’ is a random number ranging from −1 to +1.

The entire process of WOA can be summarized using pseudocode, as shown in Supplementary Material S1.

**Grey Wolf Optimizer**

A population-based meta-heuristic algorithm called GWO was proposed by Seyedali Mirjalili et al. in 2014 [53], and simulates the leadership hierarchy and hunting mechanism of grey wolves (Canis lupus) in nature. The leadership hierarchy in grey wolves is simulated by a strict social dominance hierarchy involving four types of grey wolves, namely the omega, delta, beta, and alpha. First, as per the social dominance hierarchy represented in Figure 1, the alpha α wolf is considered the pack’s leader (involving 5–12 grey wolves), and other members of his/her group follow the decision made by the alpha. Second, the betas are the next in line in the social dominance hierarchy of the grey wolves. They are the sub-ordinates to the alpha and assist the alpha in decision-making. Third, the deltas yield to the alpha and delta, but they dominate the omegas. Finally, the omega is the least essential member of the pack and is only allowed to eat the left-out food. The mathematical modeling of the social hierarchy and hunting mechanism of the GWO involves five main phases:

**Social Hierarchy**

(1) The best solution is designated as an alpha wolf (α);
(2) The second best solution is selected as a beta wolf (β);
(3) The third best candidate solution is defined as the delta wolf (δ);
(4) And the remaining solutions are considered as the omega wolves (ω).

**Searching for Prey (Exploration)**

[A] > 1 guides the grey wolves to diverge and find a better, fitter prey (exploration) and C, which is a random value ranging from 2 to 0. If C > 1 represents attacking, C < 1 does not emphasize an attack. Both A and C represent the coefficient vectors.

**Encircling the Prey**

The encircling of the prey is mathematically demonstrated using the following equations:

(19) $$\vec{D}=\left|\vec{C}.{\vec{X}}_{p}(t)-{\vec{X}}_{p}(t)\right|$$

(20) $$\vec{X}(t+1)={\vec{X}}_{p}(t)-\vec{A}.\vec{D}$$

Here, *t* signifies the existing iteration, *A* and C are the co-efficient vectors, *Xp* is the prey’s position vector, *X* denotes the position vector of the grey wolf, and *X* (*t* + 1) indicates the position vector of a grey wolf at the subsequent iteration. Moreover, the co-efficient vectors *A* and *C* are mathematically calculated using the following equations:

(21) $$\vec{A}=2\vec{a}.{\vec{r}}_{1}-\vec{a}$$

(22) $$\vec{C}=2.{\vec{r}}_{2}$$

Here, for exploring and encircling the prey, the component ‘*a*’ is linearly decreased from 2 to 0 over a set number of iterations. Based on the value of the element ‘*r*_1_ and *r*_2_’, the random vector value ranges from 0 to 1 [0, 1].

**Hunting the Prey**

At each iteration, omega wolves update their positions, comprehending the location of α, β, and δ since the α, β, and δ have a better understanding of the potential position of the prey. Therefore, the positioning of the omega wolves can be mathematically modeled using the following equations:

(23) $$D=\left|{\vec{C}}_{1}.\vec{X}(t)-\vec{X}(t)\right|\;,\;D=\left|{\vec{C}}_{2}.\vec{X}(t)-\vec{X}(t)\right|\;,\;D=\left|{\vec{C}}_{3}.\vec{X}(t)-\vec{X}(t)\right|$$

(24) $${\vec{X}}_{1}(t+1)=X(t)-{\vec{A}}_{1}.D\;,\;{\vec{X}}_{2}(t+1)=X(t)-{\vec{A}}_{2}.D\;,\;{\vec{X}}_{3}(t+1)=X(t)-{\vec{A}}_{3}.D\;$$

(25) $$\vec{X}(t+1)=\left({\vec{X}}_{1}+{\vec{X}}_{2}+{\vec{X}}_{3}\right)/3$$

**Attacking the Prey**

When the prey halts, the grey wolf ends the hunting process by assaulting the target (prey), and subsequently, the value of ‘a’ decreases. ‘*A*’ is a random value that ranges from −2a to +2a, where ‘*a*’ is reduced from 2 to 0 over the entire iterations. Finally, if |A| < 1, the wolves exploit the prey (attacking).

The entire process of the GWO algorithm can be summarized using pseudocode, as shown in Supplementary Material S2.

**Sine Cosine Algorithm**

The SCA is a recent meta-heuristic population-based optimization technique motivated by trigonometric sine and cosine functional characteristics. Although Seyedali Mirjalili et al. proposed the SCA algorithm in 2016 [54], SCA has been used to solve various optimization problems in different research fields. The SCA algorithm uses a mathematical model based on sine and cosine functions to make several preliminary arbitrary candidate solutions and requires the agents (solutions) to vary towards or away from the optimal global solution. The working process of the algorithm in terms of mathematical equations is demonstrated using the following equations:

(26) $$X_{i}^{t+1}=X_{i}^{t}+r_{1}\;\times \;\sin\;(r_{2})\;\times \;\left|r_{3}P_{i}^{t}-X_{i}^{t}\right|$$

(27) $$X_{i}^{t+1}=X_{i}^{t}+r_{1}\;\times \;\cos\;(r_{2})\;\times \;\left|r_{3}P_{i}^{t}-X_{i}^{t}\right|$$

In addition, the random variables, namely *r*_1_, *r*_2_, *r*_3_, and *r*_4_, and certain adaptive variables, namely *X^T^*, which is the location of the present solution in the *i*-th dimension and *t*-th iteration, and *Pt*, which is the destination point in the *i*-th dimension, have been integrated into this algorithm to underline the exploration and exploitation of the search space in various optimization purposes, as shown in Equation (28).

(28) $$X_{i}^{t+1}=\left\{\frac{X_{i}^{t}+r_{1}\;\times \;\sin\;(r_{2})\;\times \;\left|r_{3}P_{i}^{t}-X_{i}^{t}\right|\;,\;r4<0.5}{X_{i}^{t}+r_{1}\;\times \;\cos\;(r_{2})\;\times \;\left|r_{3}P_{i}^{t}-X_{ii}^{t}\right|\;,\;r4\geq 0.5}\right.$$

where *r*_4_ is a random number in [0, 1].

The entire working process of the SCA algorithm can be presented using pseudocode, as shown in Supplementary Material S3.

**Genetic Algorithm**

**Figure 3:**
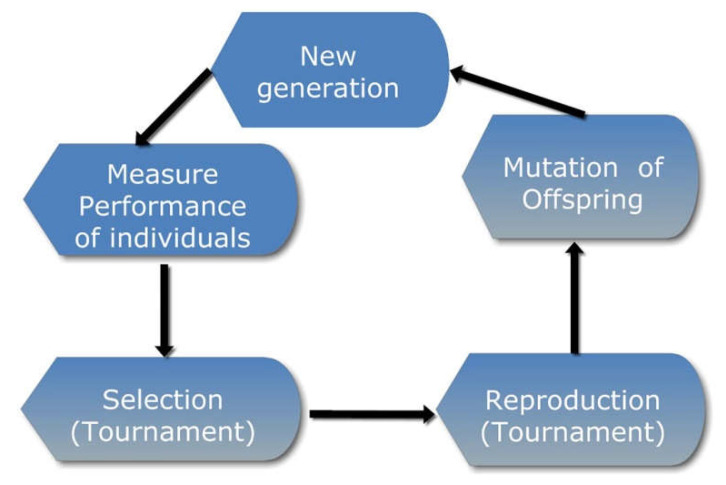
Workflow of genetic algorithm.

The GA utilizes a method to finalize an optimal set of attributes based on the concept of evolution [55,56,57]. The workflow of the GA is pictorially represented in Figure 3. First, an initial population-based on the subset of the possible attributes is generated for feature selection to select an optimal feature subset. Then, the subset is evaluated using a predictive model for performing the target task from the feature population. First, the fitness of each feature of the feature population is considered. Then, on consideration, a tournament is conducted to select the subset of features as parents for the next generation for reproduction. The next generation involves a cross-over between the tournament winners (mixing the winning attribute sets with attributes from other winners). Then, the algorithm performs mutation (introduce or delete some features randomly). A mutation is committed to maintain the variation in the population (feature sets), and thus avoid early convergence. Finally, the algorithm proceeds for a set number of iterations (generations) or terminates when the feature set has converged (the process does not produce off springs significantly different from the previous generation). Thereafter, it can be concluded that the genetic algorithm has provided the optimal feature set (population member (s)) for the provided task. The entire process of the algorithm can be summarized using pseudocode, as shown in Supplementary Material S4.

### 2.6. Training

The 80% training set was used to perform stratified five-fold training-cum-cross-validation of seven machine learning models: LR, KNN, RF, XGBoost, SVM, Gaussian-NB, and DT generated using the optimal feature subset screened using the hybrid feature selection technique. Each model built using the four optimal feature subset was evaluated to select the best performing model that can predict the outcome of the COVID-19 patient on admission with high accuracy, F1 score, and ROC-AUC value.

### 2.7. Classification Algorithms

The three supervised machine learning algorithms used for the training classification model built using the four optimal feature subset are described below:

#### 2.7.1. Logistic Regression

The LR is an interpretable generalized linear model that performs classification on small-size linearly separable data [58,59]. The LR classifies discrete class variables based on a sigmoid function, where the input variables can have a value ranging from +∞ to −∞ and the output of a probability of an event (outcome variables). The LR algorithm was trained with a ‘lbfgs’ solver owing to the small size dataset, L2 penalty, dual = false (as the n_samples > n_features in the COVID-19 dataset), the inverse of regularization strength ‘C’ was set to 1, tolerance for stopping criteria was fixed to 0.0001, maximum iteration = 100, and intercept scaling was set to 1.

#### 2.7.2. XGBoost

In the XGBoost classifier, XGBoost stands for extreme gradient boosting. The XGBoost algorithm is a scalable, optimized, distributed gradient-boost decision tree library. Moreover, the algorithm provides a parallel tree boosting and can solve classification, regression, and ranking problems quickly and accurately [60]. The XGBoost classifier was trained to develop a predictive model for predicting mortality. The XGBoost model was trained by setting objective = binary:logistic, max_depth = 4 (maximum tree depth for base learner), learning_rate = 0.2 (boosting learning rate), reg_lambda = 1 (L2 regularization term on weights), n_estimators = 150 (number of boosting rounds), subsample = 0.9 (subsample ratio of the training instance), colsample_bytree = 0.9 (subsample ratio of columns when constructing a tree), and random_state = 1 (random number seed).

#### 2.7.3. Gaussian Naïve Bayes

The GNB is a modified version of NB and assumes that the continuous data in the training data for each variable are associated with each class and are distributed as per Gaussian (or normal) distribution. The Naïve bayes algorithm was trained with the parameters (prior probabilities of the classes) set as ‘none’ and var_smoothing (portion of the largest variance of all features, which is added to variances for calculation stability) was selected as 1 × 10^−9^.

#### 2.7.4. Decision Trees

The DT is a nonparametric supervised learning algorithm used to predict the outcome variables by learning simple decision rules deduced from the training data [61,62]. The decision tree algorithm was trained with the following settings: Criterion (the function to measure the quality of a split) = ‘Gini’, splitter 9 (the strategy used to select the split at each node) = best, maximum depth (the maximum depth of the tree) = 4, min_samples_split (the minimum number of instances required to split an internal node) = 2, and min_samples_leaf (the minimum number of instances required to be at a leaf node) = 1.

#### 2.7.5. K-Nearest Neighbor

The KNN is a nonparametric supervised learning technique [63]. It solves both classification and regression data science problems. The input for the KNN algorithm involves the ‘k’ closest training instances in the training dataset in both classification and regression problems. For example, in our study, the k-nearest neighbor’s algorithm was trained with the following settings: N_neighbors (number of neighbors) = 5, weights (weight function used in prediction) = ‘uniform’, algorithm = ‘auto’, leaf_size = 30, *p* (power parameter for the Minkowski metric) = 2, and metric (the distance metric used for the tree) = Minkowski.

#### 2.7.6. Support Vector Machine

The SVM is a machine learning algorithm that distinctly classifies data points by finding an appropriate hyperplane in an n-dimensional feature space, and is helpful for small data sizes where the number of features is greater than the number of instances in the training data [64]. The SVM model was trained with the following parameters: Penalty (specifies the norm used in the penalization) = ‘L2’, loss (specifies the loss function) = ‘squared_hinge’, tol (tolerance for stopping criteria) = 0.0001, C (regularization parameter) = 1.0, fit_intercept (whether to calculate the intercept for this model) = true, intercept_scaling = 1.

#### 2.7.7. Random Forest

The RF is a robust, tree-based classification technique where an ensemble of many individual decision trees predicts a sample’s class outcome, and the class with maximum votes turns into the RF model’s outcome prediction of the sample [65]. The RF algorithm uses attribute randomness and bagging while creating each tree to develop an uncorrelated forest of decision trees, where the prediction by the forest of individual trees is substantially more accurate than any single decision tree. Moreover, the RF algorithm shows significant prediction accuracy for the small-size dataset. The RF algorithm was trained with the following parameter values: Criterion = Gini, n_estimators = 100, maximum depth = 4, min_samples_split = 2, min_samples_leaf = 1, max_features = ‘auto’, and max_leaf_nodes = none, min_impurity_decrease = 0.0, bootstrap = true, number of trees = 90, and oob_score = false.

### 2.8. Testing

The models trained using the four optimal feature subset were tested using the stratified five-fold cross-validation on the preprocessed 20% independent test. In addition, the model’s predictive performance evaluation metrics (accuracy, F1 score, and ROC-AUC value) as well as the means and standard deviation values were recorded and compared to screen the best performing model in mortality prediction.

### 2.9. Histogram Frequency Curve Plot

A histogram frequency curve depicts the frequency distribution of continuous attributes (in our case, blood biomarkers) between the two classes of population of instances from the COVID-19 dataset. In our study, the histogram frequency curve plot was employed to depict the difference between the population mean of continuous attributes and the two classes of population (non-survivor and survivor).

### 2.10. Student t-Test

We performed the two-tailed unpaired *t*-test [51] to select attributes that demonstrate a significant difference (*p*-value < 0.05) in the mean value of the features between the COVID-19 non-survivor and survivor population in the COIVD-19 dataset. Moreover, we performed a one-tailed unpaired *t*-test [49] to compare the performance of our model with the recently published machine learning models built using clinical biomarker data for an earlier prediction of COVID-19 mortality.

### 2.11. Web Application

The final ML predictive model was hosted on Heroku, a cloud-based platform, to implement a web application entirely in the cloud environment. Moreover, the output of the web application is probability score-based. For example, positive COVID-19 samples with an output probability score >0.5 have a higher probability of mortality than samples with a probability score ≤0.5.

## 3. Results

### 3.1. Identification of Novel Combination of Blood Biomarkers

In the present scenario of a rapid increase in COVID-19 and the resulting mortalities, many lab tests are required to evaluate individuals’ medical situations. Feature selection identifies the most relevant blood biomarker for reliable mortality prediction of COVID-19 individuals. Therefore, fewer blood biomarker estimations indicate more periodic lab tests, resulting in faster and more effective policymaking processes in health care management of individuals admitted with confirmed COVID-19 cases. As a result, a hybrid feature selection approach was employed to screen the imminent mortality risk blood markers. Combinations of a multivariate filter-based method, such as mRMR, student *t*-test, and four state-of-the-art nature-inspired meta-heuristic methods for global optimization, namely WOA, GA, GWO, and SCA, were employed to screen the most relevant features of COVID-19 patient mortality prediction.

#### 3.1.1. The mRMR Feature Importance

The mRMR selects a subset of features with the slightest correlation and the highest correlation with the outcome variables. The relevance and redundancy in the mRMR algorithm were calculated using the FCQ. The subset of features obtained using the mRMR algorithm is listed in Table 2.

#### 3.1.2. Student *t*-Test-Based Feature Selection

The *p*-values at a 5% significance level of the mean value difference for the 20 most informative features in Table 2 were selected using mRMR between two classes of population (survivor and non-survivor), as shown in Table 3.

Moreover, the frequency distribution histogram plot, depicting the mean frequency distribution of blood biomarkers between the two classes of population screened using mRMR, is pictorially represented in Supplementary Figure S1. The blood biomarkers, with mean population difference between the two classes of population (survivor and non-survivor) lower than < 0.5 (5% significance level), were considered significant and selected for further feature selection analysis using meta-heuristic methods. Therefore, sixteen features, with *p*-values lower than the 5% significance level, were selected and further screened to identify an optimal subset of features with a more remarkable ability to predict the mortality of individuals admitted to hospital with positive COVID-19 test results.

#### 3.1.3. Meta-Heuristic Method-Based Feature Selection

The present study presents a comprehensive analysis of nature-inspired meta-heuristic method, which is used in feature selection. Meta-heuristics are problem-independent optimization methods that iteratively explore the entire search space and assist in identifying the most informative subset of features to achieve a predictable model with a better F1 score and accuracy. The subset of features obtained using the four state-of-the-art meta-heuristic methods is listed in Table 4.

**Figure 4:**
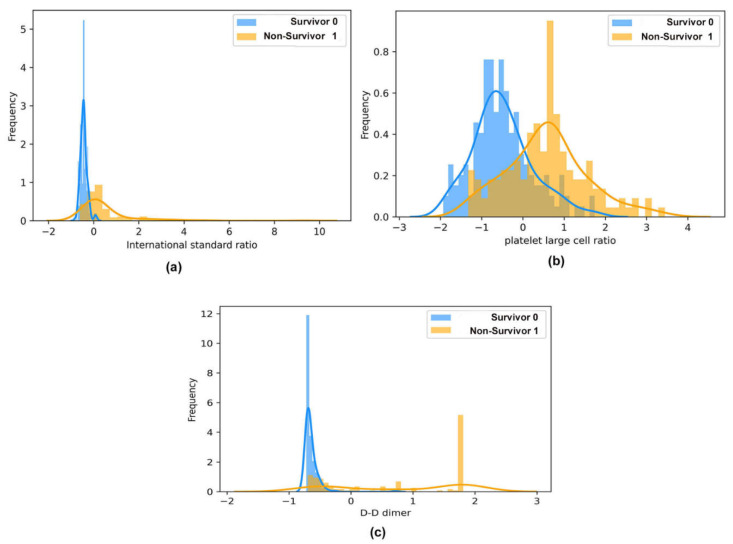
The histogram represents the frequency distribution across the two classes of population (non-survivor and survivor) of the four optimal blood biomarker subset obtained using the WOA meta-heuristic technique. (**a**) Platelet large cell ratio, (**b**) D-dimer, and (**c**) international standard ratio.

The frequency distribution of the best subset of features obtained using WOA between individuals’ two classes (survived and death) of population (survived and death) is represented using a histogram plot in Figure 4a–c. In addition, the selected blood biomarker is statistically significant (*p* < 0.001), which is verified using a two-tailed unpaired *t*-test, as shown in Table 2. Therefore, the mean distribution of the selected blood biomarkers in the two classes of population of the patient group, namely, the survived group and the death group, is statistically significant at *p* < 0.001.

**Figure 5:**
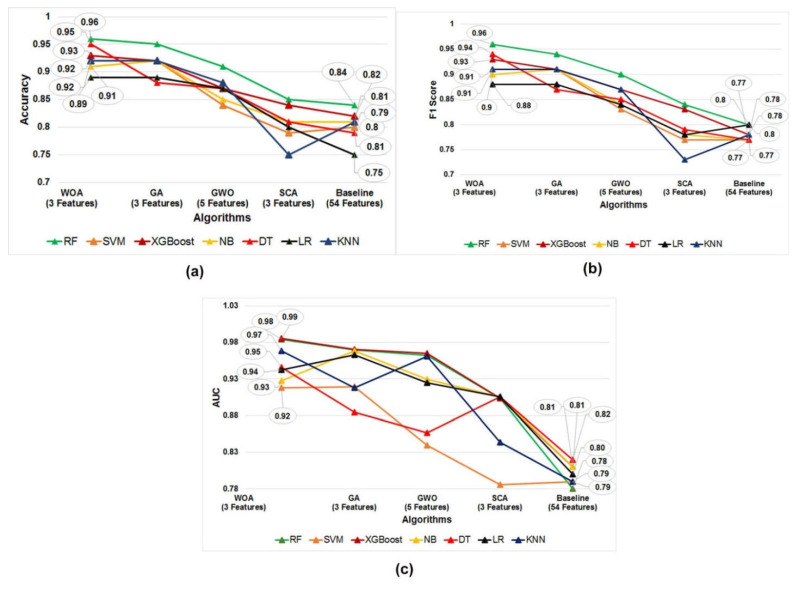
Comparison of the performance matrices. (**a**) Accuracy, (**b**) F1 score, and (**c**) AUC value of the three predictive models built using the four optimal feature subset obtained by WOA, GA, GWO, and SCA meta-heuristic algorithms.

The supervised classification algorithms, namely SVM, NB, DT, LR, KNN, RF, and XGBoost, were trained, validated, and tested on the datasets obtained using each of the four subset of features with meta-heuristic methods. In addition, stratified five-fold cross-validation was applied over all of the training and independent test datasets. We aimed to screen the most optimal subset of the blood biomarker-based classification predictive model to predict the clinical outcome of COVID-19 individuals admitted to the hospital. Figure 5a–c shows the accuracy, F1 score, and AUC of all the developed models using the features obtained using five different meta-heuristic methods.

**Figure 6:**
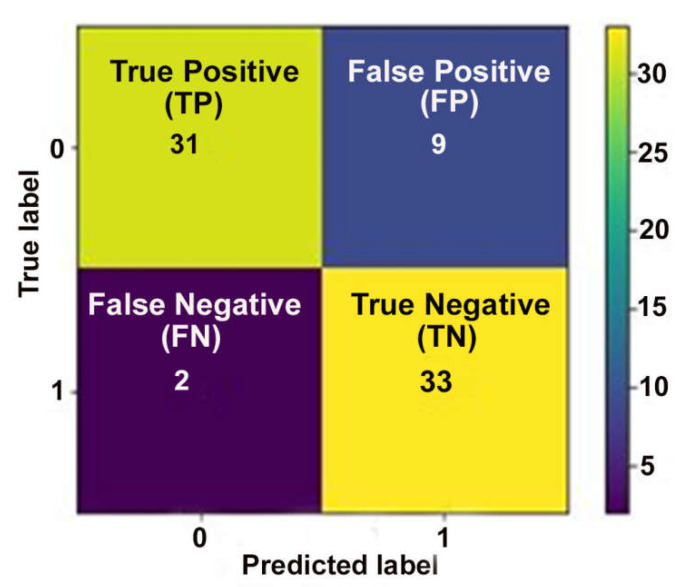
Confusion matrix of the RF-based COVID-19 mortality prediction model was tested on the 20% independent test dataset.

In accordance with the comparative performance evaluation of models, which is represented in Figure 5a–c and listed in Table 5, it was observed that the RF predictive model built using the feature subset obtained using the WOA is the best in terms of accuracy (0.96 ± 0.062), F1 score (0.96 ± 0.099), and AUC value (0.98 ± 0.024). In addition, a confusion matrix describing the performance of the RF-based predictive model on an independent test dataset is represented in Figure 6.

A summary of the baseline model performance of the eight models built using all of the features in the COVID-19 dataset is listed in Table 4. The RF-based predictive model performance estimated that accuracy, F1 score, and AUC were better than the baseline models built using all of the features and base classifiers, namely SVM, NB, DT, LR, KNN, RF, and XGBoost, as shown in Figure 5a–c and listed in Table 4. Therefore, in accordance with our results, the best subset of blood biomarkers derived from the present study is the ‘international standard ratio’, ‘platelet large cell ratio’, and ‘D-dimer. Moreover, the unpaired student *t*-test statistical analysis shows that the selected features can be used as prognostic blood biomarkers to predict the outcome of a COVID-19 patient during their stay at the hospital. Furthermore, the RF-based model built using the optimal feature subset obtained by WOA was identified as the best performing model in predicting mortality of positive COVID-19 individuals.

### 3.2. Comparative Performance of Our Model with Other Relevant Models

Our proposed RF-based predictive model was compared with the single tree XGBoost model [41], neural network (NN)-based classification model [42], and logistic regression model [32], which were built by the blood biomarkers available from the time series data generated by Yan et al. 2020. The comparative performance evaluation of the three models based on accuracy, F1 score, and AUC value is listed in Table 6. Our proposed RF-based ML model performed better in accuracy, F1 score, and AUC value than the models presented by Yan et al. 2020, Rehman et al. 2021, and Karthikeyan et al. 2021 for predicting the outcome of COVID-19 individuals on hospital admission.

### 3.3. Implementation of the Proposed Model

The RF-based ML predictive model, which predicts in-hospital mortality of COVID-19 individuals, has been successfully implemented as a web application and hosted at https://appcovid19mortality.herokuapp.com/, accessed on 25 May 2022. In our implementation, the user can send input data and receive predictions in real-time regarding the outcome of the COVID-19 patient admitted to the hospital. Therefore, our web-based application can be used by clinicians for real-time mortality risk prediction of COVID-19 individuals in the health care system, with limited healthcare facilities.

## 4. Discussion

The unexpected increase in COVID-19 cases creates an immense pressure on healthcare systems world-wide. In these times, a rapid and accurate early clinical assessment of COVID-19 individuals at high risk of mortality is a critical resource to optimize a treatment for individuals progressing to severe clinical complications. Accordingly, proper medical treatments can be administered to COVID-19 individuals when we more clearly understand the major risk factors that influence mortality. Moreover, due to the recent increase in COVID-19 infection, new clinical factors that affect the disease’s progression are continuously investigated and discovered. Therefore, ML methods can discern valuable, multidimensional clinical data patterns in the current situation. The present study reports on the screening of a novel subset of blood biomarkers to build a simple interpretable RF-based predictive application, which is anticipated to provide highly accurate support for the identification of COVID-19 individuals at high risk of mortality.

In this study, we aimed to identify a novel combination of clinical blood biomarkers and implement an ML model based on the screened biomarker for the prediction of COVID-19 individuals with high mortality risk. Herein, mRMR and the meta-heuristic-based hybrid feature selection protocol were used to filter the most informative and relevant blood biomarkers for the development of an ML-based predictive model, which precisely and quickly predicts the risk of death of COVID-19 individuals on admission to the hospital. Therefore, the hybrid feature selection method screened a novel set of three blood biomarkers for mortality prediction. The selected three features included the following blood biomarkers: International standard ratio, platelet large cell ratio, and D-dimer. Moreover, in the last 2 years, clinical studies have found that these screened blood biomarkers are potential predictors of severity and mortality of COVID-19 individuals [24,26,32,39,40,41,42,43,44,45,46,47,66].

The D-dimer is a cross-linked fibrin degradation product, which is a common biomarker for thrombotic disorders. Thrombotic complications involving increased levels of D-dimer are more common in non-survivors of COVID-19, and the D-dimer value greater than 1 μg/mL increases the odds of in-hospital death [66,67,68,69]. The authors of [70] showed that D-dimer strongly correlates with COVID-19 disease severity and is a potential biomarker for in-hospital mortality prediction of individuals suffering from COVID-19. Recently, the authors of [71] reported that 1.5 μg/mL is the optimum threshold value of D-dimer for the mortality prediction of COVID-19 individuals at the time of admission to the hospital. Therefore, they concluded that the D-dimer value at the time of COVID-19 patient admission is a reliable biomarker for in-hospital mortality prediction of confirmed COVID-19 individuals. The authors in [72] performed a systematic review and meta-analysis of 113 studies. They concluded that a rapid assessment of D-dimer levels should be conducted for COVID-19 individuals to predict the adverse outcome in individuals admitted to the hospital for treatment of COVID-19 infection. In the present study, our results also showed that the mean difference for the D-dimer population distribution in the survivor and non-survivor classes is statistically significant (*p* < 0.001) when verified using a two-tailed unpaired *t*-test *p* < 0.001. Therefore, in accordance with our results and recent clinical findings, we suggest that the large platelet cell ratio distribution can be used as a prognostic biomarker for predicting the severity and outcome of COVID-19 individuals.

Viral infections lead to platelet activation via various pathophysiological pathways, including direct interactions of the inflammatory moderators with the viral pathogen and viral antigen-antibody complexes [73]. Platelet activation, conversely, changes standard laboratory platelet indices, namely mean P-LCR, platelet volume (MPV), procalcitonin (PCT), and platelet distribution width (PDW). The P-LCR is a marker of circulating larger platelets (>12 fL) and is represented as a percentage. Moreover, the P-LCR is used as a biomarker to measure the activity of platelets. The standard percentage range is 15–35%. The PCT quantifies the total platelet mass as the percentage of volume in the blood. The standard range for PCT is 0.22–0.24%. The MPV is a primary examined platelet parameter, which implies the mean size of circulating platelets in the blood. In normal individuals, the MPV value ranges from 7.2 to 11.7 fL. An MPV value beyond 13 fL is an indicator of hypertension, while an MPV value lower than 7.2 signifies a lower production of platelets [74,75]. Changes in these standard platelet indices lead to deranged platelet parameter values, thereby signifying that platelet activation is a potential prognostic blood biomarker in multiple disease processes, including critical illness due to viral infection and venous and arterial thromboses malignancies [76]. Therefore, the role of platelet activation and the changes in the laboratory platelet indices on COVID-19 infection leading to immune thrombosis and thrombocytopenia are significant representations in non-survivors and progressive disease severity in confirmed COVID-19 cases [77,78]. The authors of [79] suggested that changes in platelet indices are potential blood biomarkers that lead to an activation of the coagulation system of COVID-19 individuals. The positive COVID-19 cases (*n* = 353) had statistically significant (at *p* < 0.001) increases in P-LCR, PDW, and MPV when compared with COVID-19 negative cases (*n* = 51). The authors of [80] showed that COVID-19 positive cases with high P-LCR (95% CI, 1.40–6.41; *p*  =  0.0046) were significantly associated with worse survivability. The authors of [81] proposed that P-LCR is the most important biomarker for evaluating platelet activity and could identify COVID-19 individuals with an increased risk of thrombotic events. In another study [82], the authors proposed the use of a cohort study, in which the values of the P-LCR parameter were significantly higher in COVID-19 individuals than in non-COVID-19 individuals. In our study, the mean difference in the P-LCR distribution of the two classes of population (survivor and non-survivor), is statistically significant (*p* < 0.001) when verified using a two-tailed unpaired *t*-test *p* < 0.001. Therefore, in accordance with our results and recent clinical findings, we suggest that the large platelet cell ratio distribution can be used as a prognostic biomarker for predicting the severity and outcome of COVID-19 individuals.

Persistent and higher INR levels correlate significantly with COVID-19 severity and mortality. INR levels are calculated by dividing the prothrombin level of a COVID-19 patient by the standardized PT levels (control). First, they are measured to assess the COVID-19-associated coagulopathy. Second, the mean INR value in patients with severe disease or non-survivor status was >1.2 (with a normal range of 0.8 to 1.1) [83]. The authors of [84] conducted a meta-analysis and systematic review of the INR in COVID-19, and found that INR and DD are highly correlated with disease severity and mortality. Therefore, it can be used as a diagnostic marker for predicting the COVID-19-associated coagulopathy and the clinical outcome of in-hospital COVID-19 individuals. In another study, Jin X. et al. 2020 [85] performed a retrospective study involving 147 confirmed COVID-19 individuals from Leishenshan Hospital, Wuhan, China. The study’s objective was to find the correlation between coagulopathy and the severity of the disease in COVID-19 clinically diagnosed individuals. The authors found that the levels of INR were significantly higher in COVID-19 individuals than the healthy controls, and the INR increased levels markedly correlated with the severity of the infection in COVID-19 individuals. In a cross-sectional study conducted with 455 COVID-19 individuals in Addis Ababa, Ethiopia, the authors [86] analyzed the coagulation profile of in-hospital COVID-19 individuals. It was discovered that higher INR levels were found in more than 50% of the individuals with severe and critical conditions. Therefore, they recommended that INR should be monitored for admitted and in-hospital COVID-19 individuals.

Moreover, a significant difference in INR level was found between the deceased and the survivor group of individuals. Therefore, the authors concluded that COVID-19 disrupts the coagulation system, and comprehensive monitoring of coagulation agents might assist in reducing severity and controlling death due to COVID-19 in the in-hospital individuals. The authors in [87] developed a severity score ranging from 0 to 10 using different blood biomarkers involving INR to assist in predicting the severity and mortality of clinically diagnosed individuals with COVID-19. The model was developed to aid clinicians in predicting the severity and mortality of COVID-19 individuals during surge periods. In recent literature, the authors in [88] used inflammatory and blood parameters to predict prognosis in admitted COVID-19 individuals. The study found that the levels of INR, prothrombin time, and activated thromboplastin time were significantly longer in the death group of COVID-19 individuals compared with the healthy control group, and the rate increased with the severity of COVID-19 infection in the in-hospital individuals. In addition, when tested using a two-tailed unpaired *t*-test at *p* < 0.00, we observed in the present study that the mean difference for the INR distribution in the two classes of population (survivor and non-survivor), is significantly different at *p* < 0.001. Therefore, in accordance with our current findings and recent studies, we can infer that INR plays a vital role in COVID-19 disease progression and mortality of positive COVID-19 individuals.

Therefore, we suggest that using the proposed hybrid feature selection method, the selected subset of three biomarkers significantly discriminates the COVID-19 survivors from the non-survivors. To sum it up, our analysis of the selected blood biomarkers correlates with the results obtained by the previous researchers, thus implementing these biomarkers may improve the reliability of the models built to accurately predict the mortality of positive COVID-19 individuals.

We compared various ML models built using the selected three features for their predictive performance. The trained models were tested on the test set with a balanced distribution of samples in both classes (survivors (43.66%) and non-survivors (56.34%)). The RF-based model performance was better than the rest of the ML models. The RF-based model predicted the mortality of COVID-19 individuals with an accuracy of 0.96 ± 0.062, an F1 score of 0.96 ± 0.099, and AUC value of 0.98 ± 0.024 on the independent test data. The RF-based model with better metric values provides strong confidence in the proposed model, and we can say that our proposed model can be recommended for clinical testing to predict the severity and risk of positive COVID-19 individuals on admission to hospital or ICU.

Moreover, in our study, the RF-based model built using the novel combination of the three most informative blood biomarkers performed significantly better in terms of accuracy and F1 score. The AUC value is compared with other models, namely the single tree XGBoost model proposed by Yan et al. 2020 [42], NN-based classification model presented by Karthikeyan et al. 2021 [41], and LR-based model proposed by Rehman et al. 2021 [32], to predict the mortality of COVID-19 individuals. For example, the single tree XGBoost model proposed by Yan et al. 2020 using LDH, hs-CRP, and lymphocytes as blood biomarkers was able to predict the mortality of COVID-19 individuals with an accuracy of 90 ± 0.537 and F1 score of 95 ± 0.06. In addition, the single tree XGBoost based model was able to differentiate between the two classes (survivor and non-survivor) with an AUC value of 97.77 ± 1.82. On the other hand, the NN-based classification model proposed by Karthikeyan et al. 2021, built using six blood biomarkers (lymphocytes, neutrophils, hs-CRP, LDH, and age), was able to predict the mortality of COVID-19 individuals with an accuracy of 96.526 ± 0.637 and F1 score of 0.9687 ± 0.006. In addition, the NN-based model can differentiate between the two classes with an AUC value of 0.9895 ± 0.0057. Furthermore, the logistic regression model proposed by Rehman et al. 2021 predicted mortality with an accuracy of 0.92 ± 0.03, F1 score of 0.93 ± 0.03, and the model differentiated the two classes with an AUC value of 0.992 ± 0.008.

Conversely, our RF-based model predicted the mortality of COVID-19 individuals with better accuracy (0.96 ± 0.062) and F1 score (0.96 ± 0.099) than recent models built using the same COVID-19 dataset. Moreover, our proposed model better classified survivors from non-survivors with an AUC value of 0.98 ± 0.024. The comparative model performance study shows that the three biomarkers selected using the hybrid selection method highly influence the predictability of an ML model to predict the mortality of COVID-19 patients.

Considering the performance attained by our proposed model, which is built using a novel combination of three blood biomarkers, we can suggest that clinicians might resort to using our most informative combination of blood biomarkers for predicting the mortality of COVID-19 individuals admitted to the hospital, and this can assist in prioritizing the treatment of COVID-19 patients with high risk of mortality. Moreover, our RF-based model-based web application that predicts the mortality of COVID-19 individuals has been successfully implemented and available on Heroku at https://appcovid19mortality.herokuapp.com/, accessed on 25 May 2022.

### Limitations

This study has a few limitations. First, the proposed model was built on COVID-19 data obtained from individuals belonging to a hospital in the region of Wuhan, China, thereby leading the model to suffer from biases, including the viral strain found in Wuhan at that particular time, patient care, and hospital resources. Second, mutations might change the progression pattern of COVID-19 infection in other people worldwide. Finally, the study has a low number of individuals, and, in this case, all of the individuals were included from the same hospital during different study periods. As a result, the findings were difficult to generalize.

## 5. Conclusions and Future Scope

In summary, using a robust hybrid feature selection method, the present study identifies a powerful combination of three blood biomarkers (INR, P-LCR, and D-dimer) for predicting the mortality of COVID-19 individuals. The biomarker selected using the WOA algorithm plays a significant role in humans’ pathophysiology of COVID-19 infection. Moreover, the selected biomarker by mRMR and other heuristic methods play a role in the pathogenesis of COVID-19 disease. However, based on these biomarker levels in the present data, the best combination of the biomarker using WOA was employed for predicting the mortality risk of COVID-19 patients. First, biomarkers in the present study have a specific threshold value above or below a certain limit, which contributes to the pathophysiology of COVID-19 infection in humans. For example, the D-dimer range in humans varies from 0.26 to 27 μg/mL. However, a plasma value >0.5 μg/mL or an increase in D-dimer levels (greater than two times the upper boundary of the normal individual) is regarded as a pathophysiological biomarker of COVID-19 infection in humans. Second, the mean INR value in patients with severe disease or in the non-survivor category was >1.2 (normal range ranging from 0.8 to 1.1). Finally, P-LCR depicts large circulating platelets (>12 fL), which is represented as a percentage. The standard percentage of P-LCR ranges from 15 to 35%. In addition, it has been used to monitor platelet activity. Furthermore, various machine learning models were developed using the powerful combination of three biomarkers to compare and predict mortality with greater accuracy and precision. Therefore, we built an RF-based ML model that can predict the mortality of COVID-19 individuals with higher accuracy and F1 score, enabling early detection, prioritizing treatment, and possibly lowering mortality rates in individuals suffering from COVID-19. The present study requires further improvement, which is assigned for future work. First, the proposed machine learning methodology is purely data-driven, thus our model’s performance may vary when implemented on different datasets. As more blood biomarker data become available, our machine learning methodology can be repeated to obtain a robust model with consistent accuracy across other datasets. Third, the present study is a single-centered, retrospective study that primarily evaluates the clinical progression and outcome of positive COVID-19 cases. Therefore, in the future, we look forward to multi-centered and large-sample-based analysis for mortality prediction of COVID-19 individuals. Second, although we had fifty-four clinical features, we only selected and built our model based on the three most informative features, which in accordance with the modeling principle, is a trade-off between having the least number of significant features and the ability of the model to provide better prediction, thereby evading overfitting.

## Figures and Tables

**Table 1:** Studies of blood biomarker-based COVID-19 mortality risk prediction.

Studies on Blood Biomarker-Based COVID-19 Mortality Risk Prediction	Blood Biomarker	Machine Learning Algorithms	Accuracy	Area Under the Curve (AUC)	F1 score
Banerjee et al. 2020 [33]	Full Blood counts	RF and Artificial Neural Network (ANN) based models	90–91%	94–95%	NA
Brinati et al. 2020 [34]	White Blood Cell (WBC) count, and the platelets, High Sensitivity C-Reactive Protein (hs-CRP), Aspartate Aminotransferase (AST), Alanine Transaminase (ALT), Gamma-Glutamyl Transferase (GGT), Alkaline Phosphatase (ALP), and Lactate Dehydrogenase (LDH) plasma levels	RF and Three-way Random Forest (TWRF) based models	82–86%	84–86%	NA
Thell et al. 2021 [35]	Eosinophils, ferritin, leukocytes, and erythrocytes	Univariate and multivariate binomial logistic regression-based models	72.3–79.4%	0.915	NA
Yang et al. 2020 [36]	Patient demographic features (age, sex, race) with 27 routine laboratory tests	Gradient boosting decision tree (GBDT)	NA	0.854	NA
Rahman et al. 2021 [37]	Age, Lymphocyte count, D-dimer, CRP, and Creatinine	LR and developed a nomogram with LR algorithm	0.91 ± 0.03	0.992 for the external validation cohort dataset	0.92 ± 0.03
Chowdhury et al. 2021 [38]	LDH, neutrophils (%), lymphocyte (%), hs-CRP, and age	Multi-tree XGBoost model and developed a nomogram using Multi-tree XGBoost	100%	0.991 for the validation cohort dataset	NA
Vaid et al. 2020 [39]	Mortality at 7 Days biomarker: Age, Anion Gap, hs-CRP, LDH, Oxygen Saturation (SpO2), Blood Urea Nitrogen (BUN), Ferritin, Red Cell Distribution Width (RDW), Diastolic Blood Pressure	XGBoost classifier-based model	NA	In external validation, the XGBoost classifier obtained an AUC-ROC of 0.88 at 3 days, 0.86 at 5 days, 0.86 at 7 days, and 0.84 at 10 days for mortality prediction	NA
Aladağ et al. 2020 [40]	Intubated patients, a Lower Glomerular filtration rate (GFR) value, and N-terminal pro-brain natriuretic peptide (NT-proBNP) values	Multiple Logistic Regression (MLR)	NA	NA	NA
Terwangne et al. 2020 [41]	Age, acute kidney injury, lymphocytes, activated prothrombin time (aPTT), and (LDH) Levels	Bayesian network analysis for severity classification of COVID-19	NA	83.8% AUC obtained from Bayesian network trained and evaluated using the entire set of patients	NA
Huang et al. 2020 [42]	Epidemiological exposure histories, weakness/fatigue, heart rate <100 beat/min, bilateral pneumonia, neutrophil count ≤ 6.3 × 109/L, eosinophil count ≤ 0.02 × 109/L, glucose ≥ 6 mmol/L, D-dimer ≥ 0.5 mg/L, and CRP <5 mg/L	Multivariate logistic regression model based novel risk score	NA	0.921	NA
Cia et al. 2020 [43]	LDH, Neutrophil to Lymphocyte Ratio (NLR), D-dimer, and CRP score on admission and severity of COVID-19 infection	LR model	NA	The AUC values for NLR were 0.716, 0.650 for D-dimer, 0.612 for CT score, and 0.740 for LDH, which indicate a specific diagnostic value for the severity of COVID-19 infection	NA
Wang et al. 2020 [44]	The clinical model was developed using a history of hypertension, age, and coronary heart disease, and the laboratory model was developed using peripheral capillary oxygen saturation, neutrophil, hs-CRP, D-dimer, lymphocyte count, GFR, AST, and age	Stepwise Akaike information criterion and ensemble XGBoost (extreme gradient boosting) model	NA	Clinical model AUC values were 0.88 and 0.98 for the laboratory model	NA
Xie et al. 2020 [45]	LDH, age, SpO2, and Lymphocyte Count	Multivariable logistic regression model and developed a nomogram using Multivariable logistic regression	NA	Independent validation cohort with an AUC of 0.98	NA
Bolourani et al. 2021 [24]	Body mass index (BMI), age, and hypertension, to build a mortality prediction model from COVID-19 data from the United Kingdom and Denmark	XGBoost model	0.919	0.77	NA
Jimenez-Solem et al. 2021 [26]	BMI, age, and hypertension	RF-based model	NA	The model showed a higher discriminative power with an AUC of 0.818 at hospital admission, 0.906 at diagnosis, and 0.721 during ICU admission	NA
Karthikeyan et al. 2021 [46]	Neutrophils, lymphocytes, LDH, hs-CRP, and age	XGBoost feature importance and neural network classification	96.526 ± 0.637	0.9895 ± 0.0057	0.9687 ± 0.006
Yan et al. 2020 [47]	LDH, hs-CRP, and lymphocyte count	Interpretable single tree XGBoost model	NA	Predict the mortality of COVID-19 individuals with 94% accuracy as early as 3 days before the patient outcome	NA

**Table 2:** List of informative attributes from the COVID-19 clinical dataset using the mRMR algorithm.

Sl.no.	Clinical Attributes
1	Serum chloride
2	Monocytes (%)
3	Serum sodium
4	Serum potassium
5	Calcium
6	Corrected calcium
7	Indirect bilirubin
8	Prothrombin Time (PT)
9	Total Protein (TP)
10	Neutrophils (%)
11	Basophil count (BC)
12	High sensitivity C-reactive protein (hs-CRP)
13	Hemoglobin
14	International Standard Ratio (INR)
15	Platelet Large Cell Ratio (P-LCR)
16	Mean Platelet Volume (MPV)
17	Procalcitonin (PCT)
18	D-Dimer
19	Platelet Distribution Width (PDW)
20	Serum Glutamic-Pyruvic Transaminase (SGPT)

**Table 3:** List of features (blood biomarkers) obtained using mRMR and the corresponding mean difference between two classes of population (survivor and non-survivor) at a significance level of 0.5.

Sl.no.	Name of Blood Biomarkers	Mean and Standard Deviation of Blood Biomarkers between Two Classes of Population (Survivor and Non-Survivor)		Two-Tailed *p*-Value of the Mean Difference for the Blood Biomarkers between Two Classes of Population (Survivor and Non-Survivor)
		Non-Survivor	Survivor	
1	Serum chloride	0.448291385 ± 0.155	0.3763732 ± 0.119	*p* < 0.0001
2	Monocytes (%)	0.017486858 ± 0.007	0.011148923 ± 0.051	*p* < 0.0001
3	serum sodium	0.3911567 ± 0.166	0.325273619 ± 0.122	*p* < 0.0001
4	Serum potassium	0.255000148 ± 0.140	0.234565716 ± 0.070	0.0709
5	Calcium	0.556278701 ± 0.119	0.64440659 ± 0.125	*p* < 0.0001
6	Corrected calcium	0.587374724 ± 0.131	0.625911132 ± 0.104	0.0018
7	Indirect Bilirubin	0.129711839 ± 0.125	0.111754649 ± 0.096	0.1199
8	Prothrombin Time (PT)	0.089973693 ± 0.102	0.055744667 ± 0.010	*p* < 0.0001
9	Total protein (TP)	0.58374466 ± 0.162	0.648346818 ± 0.147	*p* < 0.0001
10	Neutrophils (%)	0.902449663 ± 0.097	0.757378019 ± 0.177	*p* < 0.0001
11	Basophil count (#)	0.186996944 ± 0.183	0.179833248 ± 0.163	0.6907
12	High sensitivity C-Reactive Protein (hs-CRP)	0.398503 ± 0.238	0.036965 ± 0.078	*p* < 0.0001
13	Hemoglobin	0.668481 ± 0.143	0.686667 ± 0.118	0.1821
14	International Standard Ratio (INR)	0.069874 ± 0.095	0.018222 ± 0.007	*p* < 0.0001
15	Platelet Large Cell Ratio (P-LCR)	0.513142 ± 0.179	0.414974 ± 0.178	*p* < 0.0001
16	Mean Platelet Volume (MPV)	0.482952 ± 0.184	0.383799 ± 0.178	*p* < 0.0001
17	Procalcitonin (PCT)	0.037908 ± 0.102	0.018682 ± 0.073	0.0366
18	D-Dimer	0.571878041 ± 0.408	0.280508629 ± 0.085	*p* < 0.0001
19	Platelet Distribution Width (PDW)	0.393129813 ± 0.204	0.222059325 ± 0.112	*p* < 0.0001
20	Serum Glutamic-Pyruvic Transaminase (SGPT)	0.034611327 ± 0.091	0.016735918 ± 0.014	0.0070

**Table 4:** A list of features was obtained using the four state-of-the-art meta-heuristic methods.

Meta-Heuristic Methods	Global Optimal Feature Subset
WOA	‘INR’, ‘P-LCR’, ‘D-Dimer’
GA	hsCRP’, ‘SGPT’, ‘INR’
GWO	‘Monocytes (%)’, ’TP’, ‘INR’, ‘D-Dimer’, ‘PDW’
SCA	‘TP’, ‘INR’, ‘PDW’

**Table 5:** Comparative performance evaluation of the seven predictive models built in terms of (a) accuracy, (b) F1 score, and (c) AUC value using the four optimal feature subset obtained by the WOA, GA, GWO, and SCA meta-heuristic algorithms.

**Accuracy**							
	**RF**	**SVM**	**XGBoost**	**NB**	**DT**	**LR**	**KNN**
**WOA**	0.96 ± 0.062	0.92 ± 0.024	0.93 ± 0.047	0.91 ± 0.025	0.95 ± 0.037	0.89 ± 0.053	0.92 ± 0.024
**GA**	0.95 ± 0.024	0.92 ± 0.034	0.92 ± 0.019	0.92 ± 0.027	0.88 ± 0.034	0.89 ± 0.029	0.92 ± 0.034
**GWO**	0.91 ± 0.044	0.84 ± 0.039	0.87 ± 0.032	0.85 ± 0.032	0.87 ± 0.027	0.87 ± 0.036	0.88 ± 0.039
**SCA**	0.85 ± 0.040	0.79 ± 0.045	0.84 ± 0.034	0.81 ± 0.049	0.81 ± 0.037	0.8 ± 0.049	0.75 ± 0.045
**Base line**	0.84 ± 0.044	0.80 ± 0.032	0.81 ± 0.019	0.82 ± 0.027	0.82 ± 0.036	0.75 ± 0.025	0.81 ± 0.024
**F1 score**							
	**RF**	**SVM**	**XGBoost**	**NB**	**DT**	**LR**	**KNN**
**WOA**	0.96 ± 0.099	0.91 ± 0.034	0.93 ± 0.060	0.9 ± 0.036	0.94 ± 0.048	0.88 ± 0.053	0.91 ± 0.034
**GA**	0.94 ± 0.024	0.91 ± 0.035	0.91 ± 0.017	0.91 ± 0.035	0.87 ± 0.037	0.88 ± 0.031	0.91 ± 0.035
**GWO**	0.9 ± 0.064	0.83 ± 0.052	0.87 ± 0.038	0.84 ± 0.043	0.85 ± 0.034	0.84 ± 0.053	0.87 ± 0.052
**SCA**	0.84 ± 0.058	0.77 ± 0.062	0.83 ± 0.040	0.78 ± 0.064	0.79 ± 0.047	0.78 ± 0.075	0.73 ± 0.062
**Base line**	0.80 ± 0.034	0.77 ± 0.017	0.78 ± 0.036	0.77 ± 0.053	0.77 ± 0.035	0.80 ± 0.064	0.78 ± 0.058
**AUC Value**							
	**RF**	**SVM**	**XGBoost**	**NB**	**DT**	**LR**	**KNN**
**WOA**	0.98 ± 0.024	0.92 ± 0.004	0.99 ± 0.015	0.93 ± 0.009	0.95 ± 0.011	0.94 ± 0.020	0.97 ± 0.004
**GA**	0.97 ± 0.026	0.92 ± 0.027	0.97 ± 0.015	0.97 ± 0.015	0.88 ± 0.024	0.96 ± 0.030	0.92 ± 0.027
**GWO**	0.96 ± 0.020	0.84 ± 0.025	0.97 ± 0.024	0.93 ± 0.020	0.86 ± 0.050	0.93 ± 0.014	0.96 ± 0.025
**SCA**	0.90 ± 0.052	0.79 ± 0.050	0.90 ± 0.025	0.91 ± 0.025	0.91 ± 0.025	0.91 ± 0.054	0.84 ± 0.050
**Base line**	0.78 ± 0.004	0.79 ± 0.015	0.81 ± 0.027	0.81 ± 0.011	0.82 ± 0.027	0.80 ± 0.026	0.79 ± 0.025

**Table 6:** Comparative performance evaluation between models built using the same test dataset.

Sl.no.	Author	Machine Learning Model	Blood Biomarker (Features)	Accuracy (%)	F1 score	AUC Value
1	Yan et al. 2020 [42]	Single tree XGBoost model	LDH, hs-CRP, and lymphocytes	90 ± 0.537	95 ± 0.06	97.77 ± 1.82
2	Karthikeyan et al. 2021 [41]	Neural Network (NN)-based classification model	Lymphocytes, Neutrophils, hs-CRP, LDH, and age	96.526 ± 0.637	0.9687 ± 0.006	0.9895 ± 0.0057
3	Rehman et al. 2021 [32]	LR model	Age, Lymphocyte count, D-dimer, CRP, and Creatinine	0.92 ± 0.03	0.93 ± 0.03	0.992 ± 0.008
4	Our Proposed RF-based model	RF model	INR, P-LCR, and D-dimer	0.96 ± 0.062	0.96 ± 0.099	0.98 ± 0.024

## Data Availability

Datasets are publicly available at: https://github.com/HAIRLAB/Pre_Surv_COVID_19/tree/master/data, accessed on 1 March 2022.

## Supplementary Materials

The following supporting information can be downloaded at: https://www.mdpi.com/article/10.3390/diagnostics12071604/s1. Supplementary Material S1. Whale Optimization Algorithm (WOA) pseudocode; Supplementary Material S2. Grey Wolf Optimizer (GWO) pseudocode; Supplementary Material S3. Sine Cosine Algorithm (SCA) pseudocode; Supplementary Material S4. Genetic Algorithm (GA) pseudocode; Supplementary Figure S1: The frequency distribution histogram plot depicting the mean of the frequency distribution of blood biomarkers between the two different class populations screened using mRMR.
