# Supplementary material for: A Hybrid Feature Selection Approach to Screen a Novel Set of Blood Biomarkers for Early COVID-19 Mortality Prediction
Source: Diagnostics (Basel). 2022 Jun 30;12(7):1604. doi: 10.3390/diagnostics12071604 (PMC9316550; doi:10.3390/diagnostics12071604)
Supplement: Supplementary file 1 [file diagnostics-12-01604-s001.zip › diagnostics-1769429-supplementary.pdf]

Supplementary Material

Title: A Hybrid Feature Selection Approach to Screen a Novel Set of Blood Biomarkers for an Early COVID-19 Mortality Prediction

Supplementary Material S1

Whale Optimization Algorithm (WOA) pseudocode

$Begin$

Initialize $X_i\left(i = 1, 2, 3, \ldots n\right)$, where X_i is whale population, i.e., total blood biomarker of the COVID-19 dataset

Calculate and evaluate the fitness value of whales (blood biomarkers) and select the best search agent X^* (best subset of blood biomarkers)

While $t = 1$ and $t < t_{max}$ (where t_{max} is maximum iteration)

for each blood biomarker

Update a, A, C, *l* and p [equation (14) – (18) from Manuscript]

if $(p < 0.5)$ Shrinking Encircling mechanism

if $abs(A) \geq 1$:

update the current biomarker by

$X(t+1) = X_{rand} - AD(X_{rand} = \text{Any random blood biomarker from the total blood biomarkers in the dataset})$

else if

$$X(t+1) = X^*(t) - AD$$

end if

else if $(p \geq 0.5)$ the Spiral-Shaped Attack mechanism

update the blood biomarker by

$$\vec{X}(t+1) = \overrightarrow{D'} e^{\boldsymbol{bl}} \boldsymbol{cos}(2\Pi l) + \overrightarrow{X^*}(t)$$

End if

End for

Check the better solution (subsets of biomarkers); if the optimal solution $X(t+1)$, then update X^*

End While

Return X^*

<u>Supplementary Material S2</u>

Grey Wolf Optimizer (GWO) pseudocode

*Step*1 :*Randomly initialize the population of* blood biomarkers Xi $\left(i = 1, 2, \ldots, n\right)$

*Step*2 :*Initialize the value of* $a = 2,\ A,$ *and* C $\left(\text{using eq.21 and 22 from the manuscript}\right)$

*Step*3 :*Calculate the fitness of each* biomarkers *from the* total *population of blood* biomarkers

- $X\alpha$ = *member with the best fitness value*
- $X\beta$ = *second − best member* (*in terms of fitness value*)
- $X\delta$ = *third − best member* (*in terms of fitness value*)

*Step*4 :*FOR* $t = 1$ *to Max _ number _ of _ iterations* :

- *Update the position of all the omega wolves by eq.* 23, 24 *and* 25 *from the manuscript*
- *Update* $a,\ A,\ C$ (*using eq.* 21 and 22)
- $a = 2\left(1 - t / T\right)$
- *Calculate fitness of all search agents* (blood biomarkers)
- *Update* $X\alpha,\ X\beta,\ X\delta.$

.*END FOR*

*Step*5 :*return* $X\alpha$ (best subset of blood biomarkers)

Supplementary Material S3

Sine Cosine Algorithm (SCA) pseudocode

Initialize *a set of search agents* (blood biomarkers) ***(X)***

Do

Evaluate each of the blood biomarkers ***by the objective function***

update the best blood biomarker *obtaineds so far* $\boldsymbol{(P\ (best\ solution) = X')}$

update r_1, r_2, r_3 ***and*** r_4 *(random parameters)*

update the combination of blood biomarkers using *Eq.*$(26 - 28)$ ***from the manuscript***

While $(t <$ ***maximum number of iterations)***

Return the best *subset of* blood biomarkers ***obtained so far as the global optimum***

<u>Supplementary Material S4</u>

Genetic Algorithm (GA) pseudocode

```
begin
    Create random population (subset of blood biomarkers) :
    Evaluate the fitness of each blood biomarker of the total biomarkers
    Do{
                Selection (find survival of individual blood biomarker)
                Crossover (new subset of blood biomarker generation)
                If (there is same blood biomarker)
Mutation (change new blood biomarkers of different individuals)
                        End
                        Compute fitness
                  }
                Until cover all subset of blood biomarkers
Return best individual blood biomarkers
```

Supplementary Figure S1

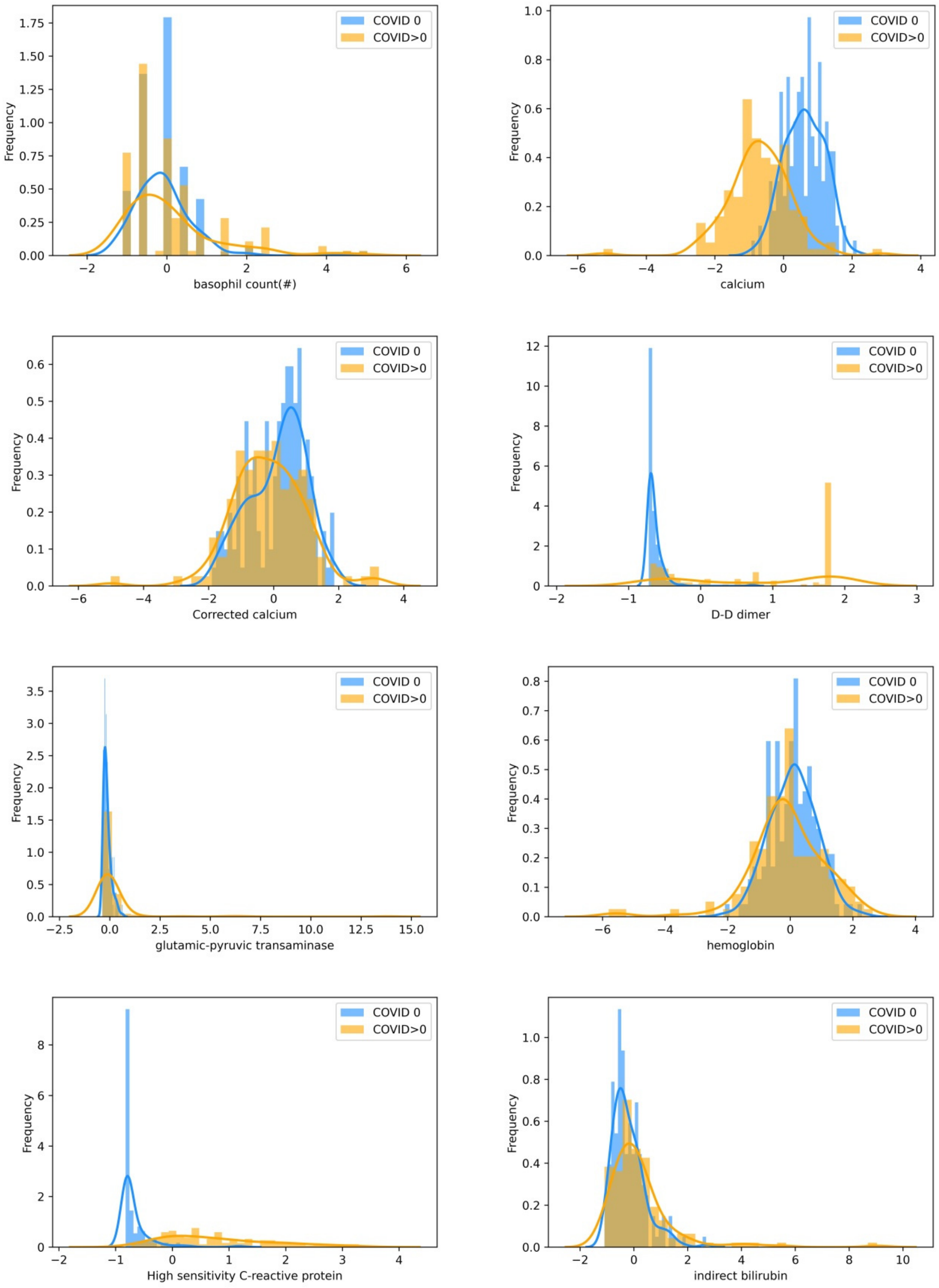

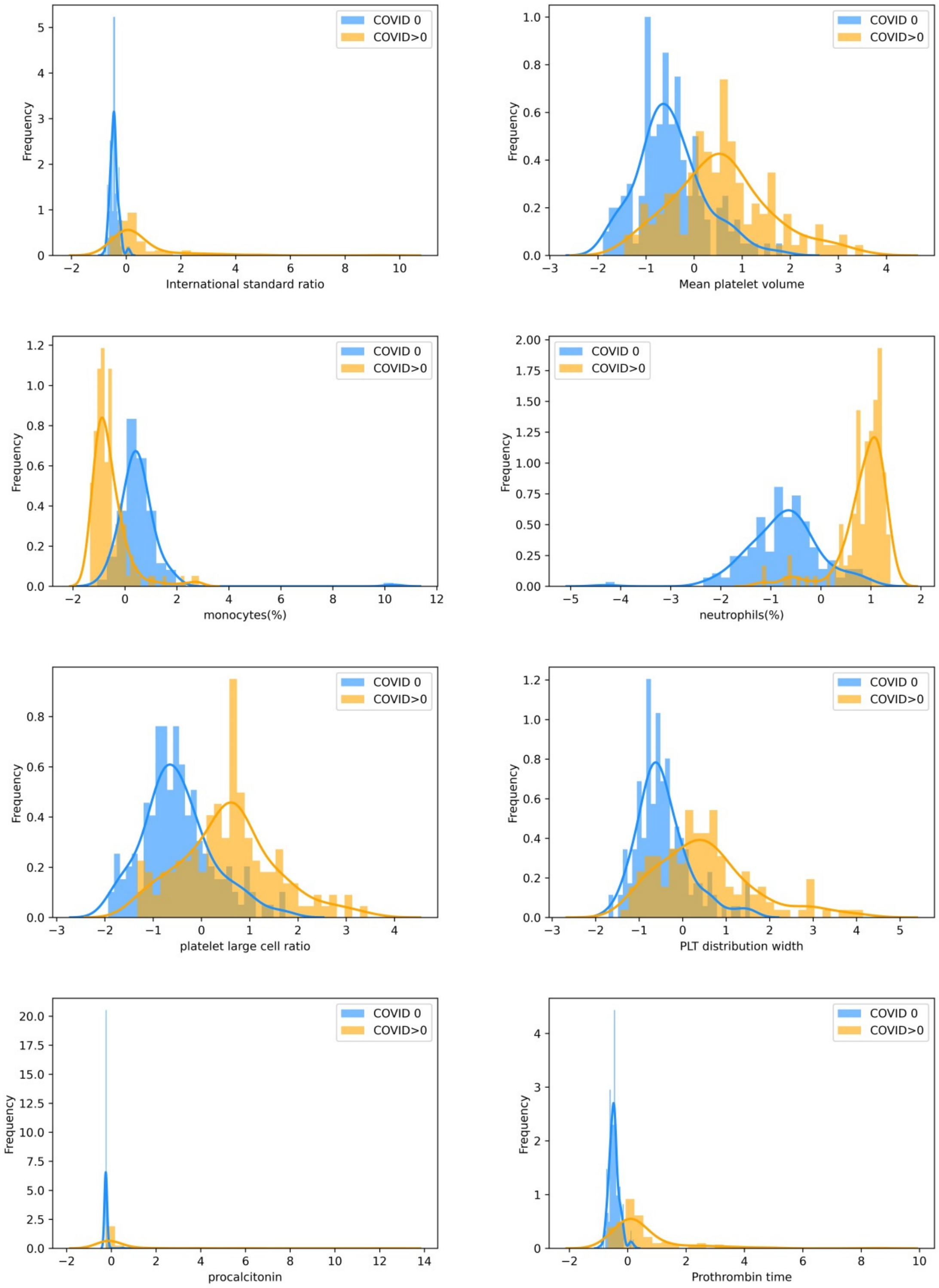
COVID 0
COVID>0
Frequency
International standard ratio
Mean platelet volume
monocytes(%)
neutrophils(%)
platelet large cell ratio
PLT distribution width
procalcitonin
Prothrombin time

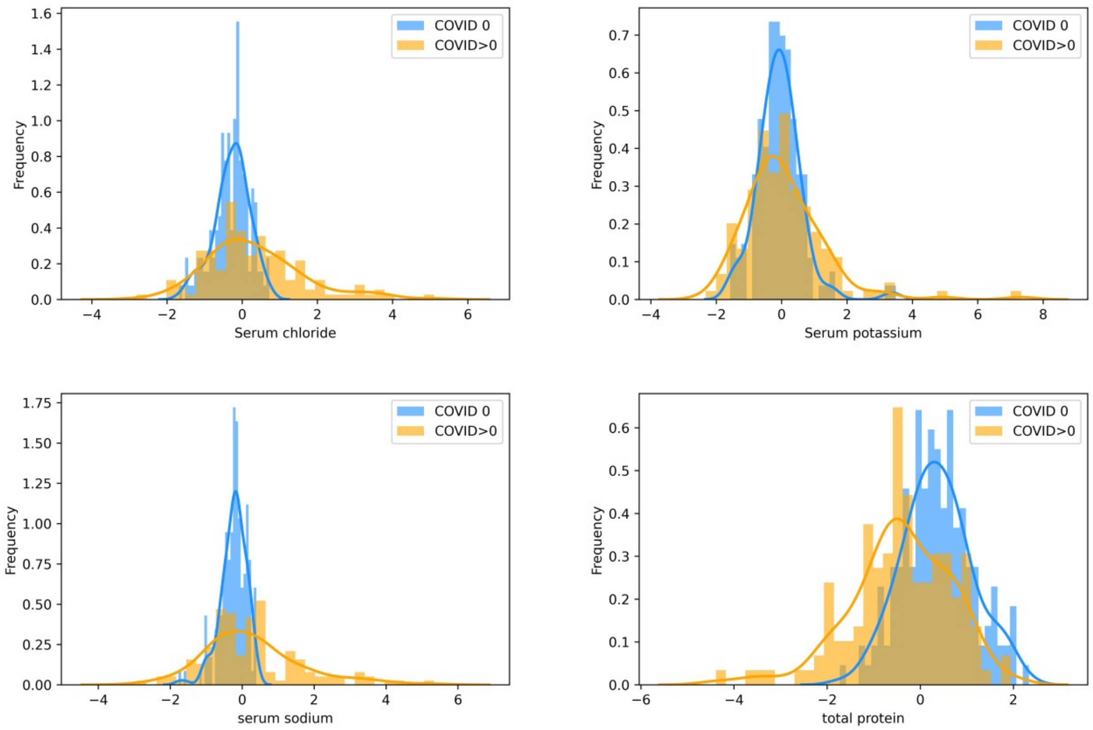

Supplementary Figure S1: The frequency distribution histogram plot depicting the mean of the frequency distribution of blood biomarkers between the two different class populations screened using mRMR.
